# Successful management of coagulation dysfunction in a patient with fulminant myocarditis: a case report

**DOI:** 10.3389/fcvm.2024.1538728

**Published:** 2025-01-13

**Authors:** Shanshan Dong, Qi Peng, Kai Lu, Qimei Wei, Jun Yang

**Affiliations:** ^1^ Department of Clinical Laboratory, Wuhan Asia Heart Hospital, Wuhan, China; ^2^ Department of Cardiac Critical Care Medicine, Wuhan Asia Heart Hospital, Wuhan, China

**Keywords:** fulminant myocarditis, cardiogenic shock, acute liver injury, coagulation dysfunction, anticoagulation, case report

## Abstract

Fulminant myocarditis (FM) is an acute, diffuse inflammatory myocardial disease characterized by abrupt onset and extremely rapid progression. Patients typically exhibit haemodynamic abnormalities that may lead to respiratory failure, liver and renal failure, and subsequent coagulopathy. Collectively, these complications significantly increase the risk of early mortality. Currently, there is limited research on coagulation dysfunction associated with FM; therefore, achieving a rebalancing of the coagulation system is a challenge for successful treatment. We report a case of coagulation disorder secondary to FM, in which the patient recovered successfully and was discharged following comprehensive treatment and correction of coagulation function. By analyzing the etiology of this condition and emphasizing strategies for correcting coagulation disorders, we aim to provide valuable references for clinical diagnosis and management.

## Introduction

Fulminant myocarditis (FM) is a specific form of inflammatory myocardial disease characterized by a complex pathogenesis involving the overactivation of cardiac innate immunity and the subsequent development of an inflammatory storm (1). The etiological factors contributing to this condition can be categorized into infectious and non-infectious agents, with viral infections identified as the predominant cause. Common viruses associated with myocarditis include enteroviruses, particularly coxsackievirus group B, as well as adenovirus, parvovirus B19, human herpesvirus 6, and COVID-19 (2, 3). Currently, the most extensively studied mechanism pertains to myocarditis induced by coxsackievirus B3 infection. The primary pathogenic mechanisms involve both direct viral damage to myocardial cells and immune-mediated damage, which ultimately lead to myocardial cell dysfunction and impaired contractility (4). As the disease progresses to FM, patients may experience severe hemodynamic instability, arrhythmias, respiratory failure, and other critical complications. Therefore, it is imperative that the patient's symptoms and clinical signs are meticulously evaluated and treated promptly. Notably, if the critical phase is successfully navigated, the long-term prognosis tends to improve. The cornerstone of managing FM includes the application of mechanical circulatory support devices to maintain hemodynamic stability and ensure adequate organ perfusion (1). Additionally, combined immune support therapy, which involves administering appropriate doses of glucocorticoids and immunoglobulins, is essential for effectively modulating immune responses (5).

Clinically, viral myocarditis may present as acute heart failure, ventricular arrhythmias, or cardiogenic shock, and it is associated with significant morbidity and mortality (6). The hypoperfusion and venous congestion resulting from cardiogenic shock can lead to damage and dysfunction in multiple target organs, including the heart, lungs, kidneys, liver, intestines, and brain (7). Among these, liver dysfunction is a critical contributor to coagulation dysfunction, as the liver is responsible for synthesizing most coagulation factors. Impaired liver function disrupts the synthesis of these factors, resulting in an imbalance between procoagulant and anticoagulant mechanisms, which can precipitate either bleeding or thrombotic events (8). Moreover, severe liver dysfunction often results in thrombocytopenia, which can occur due to several mechanisms, including increased platelet destruction from hypersplenism, diminished hepatic synthesis of thrombopoietin, and an inadequate response from the bone marrow (9). This thrombocytopenia further increases the risk of bleeding complications.

Consequently, maintaining coagulation balance through appropriate anticoagulation and blood product supplementation poses a significant challenge and is a critical aspect of managing these patients. In this report, we present a case of cardiogenic shock and acute liver injury secondary to FM, which resulted in coagulation dysfunction. The patient was effectively treated following a comprehensive therapeutic approach.

## Case presentation

A 51-year-old male patient presented to a local hospital for treatment after experiencing intermittent abdominal distension for over 10 days, accompanied by breathlessness and fatigue for the preceding 5 days. Upon hospitalization, the clinician diagnosed him with cholecystitis and initiated a course of anti-inflammatory and anti-infective therapy, which was administered for 5 days. Despite this intervention, the patient's symptoms worsened, manifesting as chest tightness, cough, dyspnea, lower limb edema, and orthopnea. As a result, the clinician expanded the diagnosis to include dilated cardiomyopathy, cardiogenic shock, atrial flutter, pleural effusion, peritoneal effusion, and abnormal liver and renal function. The treatment regimen was subsequently modified to incorporate intermittent non-invasive ventilator-assisted ventilation and symptomatic support aimed at increasing blood pressure, improving cardiac function, controlling the ventricular rate, and maintaining homeostasis; however, the patient's symptoms did not exhibit significant improvement. Consequently, he was transferred to our hospital, a tertiary cardiac specialty facility, on February 25, 2023, where he was diagnosed with heart failure, cardiomyopathy, and cardiogenic shock. Upon taking the patient's medical history, he denied any history of hypertension, diabetes, stroke, hepatitis, typhoid fever, or schistosomiasis, and he reported no known drug or food allergies. Regarding his family history, the patient's father is deceased, with the cause of death unknown, while his mother is alive and has no known cardiovascular disease.

Upon admission, the patient was conscious and exhibited mild scleral jaundice, diminished breath sounds bilaterally, and wet rales in the lower lung fields. Auscultation revealed no murmurs at any of the heart valves. Laboratory results indicated elevated cardiac biomarkers, specifically high-sensitivity cardiac troponin I (hscTnI) at 0.694 ng/ml, myoglobin at 219 ng/ml, and N-terminal pro B-type natriuretic peptide (NT-proBNP) at 5,682 pg/ml. Additionally, liver and renal function tests demonstrated impairment, with albumin at 33 g/L, alanine aminotransferase (ALT) at 5,358.2 U/L, aspartate aminotransferase (AST) at 16,734.6 U/L, lactate dehydrogenase (LDH) at 13,134 U/L, total bilirubin (TBIL) at 54.9 μmol/L, direct bilirubin (DBIL) at 21.2 μmol/L, and creatinine at 193 μmol/L. Inflammatory markers were significantly elevated, with a white blood cell count of 14.2 × 10^9^ /L, procalcitonin at 0.27 ng/ml, interleukin-6 at 33.2 pg/ml, interleukin-8 at 238 pg/ml, and high-sensitivity C-reactive protein at 14.4 mg/L. Furthermore, coagulation-related markers indicated dysfunction, with prothrombin time (PT) at 60.7 s, activated partial thromboplastin time (APTT) at 35.6 s, fibrinogen at 1.0 g/L, D-dimer at 104.5 µg/ml, thrombin-antithrombin complex at 116.5 ng/ml, antithrombin III at 43.0%, and platelet count (PLT) at 52 × 10^9^ /L.

The electrocardiogram indicated sinus rhythm with frequent premature ventricular contractions, poor precordial R-wave elevation, T-wave abnormalities, and a prolonged QT interval. Echocardiographic findings revealed a left ventricular ejection fraction (LVEF) of 20%. Both the left atrium (4.0 cm) and left ventricle (6.0 cm) were enlarged, and there was a general reduction in septal and left ventricular wall motion. The findings also indicated mild aortic regurgitation, severe mitral regurgitation, moderate tricuspid regurgitation, and markedly reduced left ventricular systolic function. Additionally, a small amount of fluid was noted in the pericardial cavity, along with a significant accumulation of fluid in the right pleural cavity. Based on the clinical signs and symptoms, electrocardiographic changes, elevated markers of myocardial injury, and echocardiographic evidence of myocardial damage observed after admission, the patient was comprehensively assessed and diagnosed with FM. Given the patient's history of antecedent infection, further testing for antibodies to myocarditis-associated viruses was conducted. The results were positive for IgM antibodies to coxsackievirus B3 and echovirus. Therefore, the patient's FM was attributed to a viral infection, which subsequently led to cardiogenic shock and severe coagulopathy.

Clinical management in this case focuses on three primary areas, the first of which is the improvement of organ function. Cardiac volume overload was addressed through negative volume balance, maintaining a deficit of 2,000 ml per day for the first four days. Cardiogenic shock was managed with norepinephrine at a dose of 0.15 µg/kg/min and dopamine at 5 µg/kg/min. An intra-aortic balloon pump (IABP) was implanted on day four of admission to enhance cardiac output and improve organ perfusion; it was withdrawn after four days of support. Amiodarone hydrochloride was administered at a rate of 30 mg/h to control arrhythmias and manage atrial flutter. Additional treatments included liver protection with reduced glutathione (1.8 g/day), diuresis, and supportive therapy such as oxygen therapy. The second area of focus was the correction of coagulation disorders. Based on laboratory findings, an International Society on Thrombosis and Haemostasis (ISTH) dominant disseminated intravascular coagulation (DIC) score of 7 (threshold >5) was obtained, suggesting a suspicion of DIC. However, the addition of a factor VIII (FVIII) activity of 176.5% and an APTT of 35.6 s did not support the diagnosis of DIC. Consequently, we initiated anticoagulation with unfractionated heparin, starting at a dose of 400 U/h, which was subsequently adjusted to 300 U/h for one week, along with continuous monitoring of D-dimer levels. Anticoagulation was accompanied by replacement therapy, including the infusion of fresh frozen plasma (FFP), with 200 ml administered on day two and another 200 ml on day five. Supplemental fibrinogen was infused, totaling 12 units of cryoprecipitate and 0.5 g of fibrinogen, to maintain fibrinogen levels above 1.2 g/L and prevent bleeding. For platelet replacement, 1 unit of platelets was infused when the platelet count dropped to 18 × 10^9^ /L, with continuous monitoring. The third area of focus was the control of inflammation and infection. Management included intravenous human immunoglobulin (PH4) at a dose of 10 g/day for five days to provide immunosupportive therapy, alongside low-dose methylprednisolone at 40 mg for five days as an anti-inflammatory treatment.

**Figure 1:**
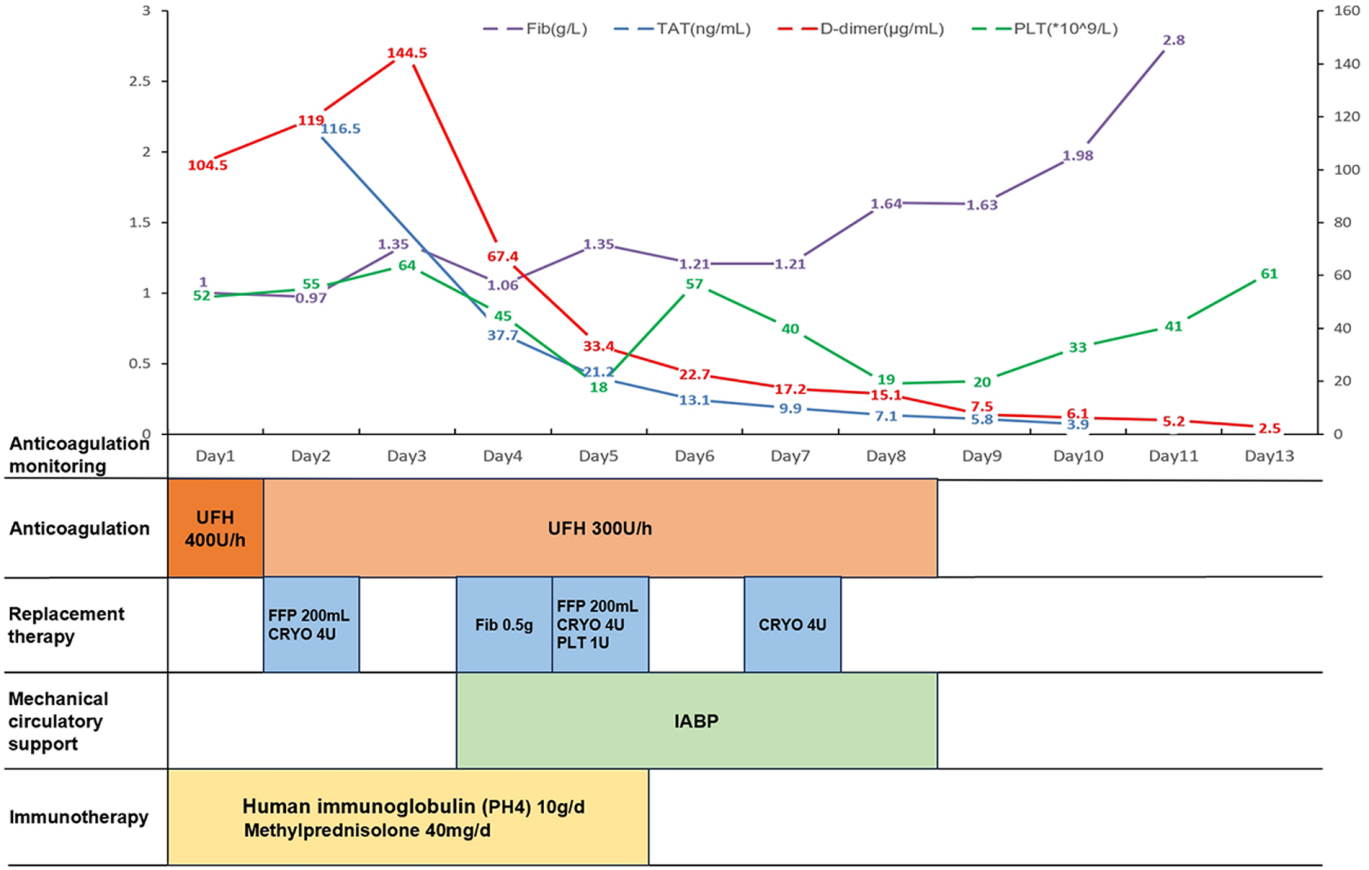
Chronological framework for anticoagulation therapy and monitoring. Fib, fibrinogen; TAT, thrombin-antithrombin complex; PLT, platelet count; UFH, unfractionated heparin; FFP, fresh frozen plasma; CRYO, cryoprecipitate; IABP, intra-aortic balloon pump.

After comprehensive treatment, the patient's organ function gradually improved, as evidenced by significantly better examination indices (Table 1). Echocardiography revealed a LVEF of 43%, with normal left atrial and left ventricular dimensions. Additionally, there was a reduced amplitude of septal motion, while the amplitude of left ventricular wall motion remained normal. Coagulation monitoring indicated that coagulation parameters had been corrected (Figure 1), with results showing fibrinogen at 2.8 g/L, D-dimer at 2.5 µg/ml, PLT at 61 × 10^9^ /L, PT at 12 s, and APTT at 27.9 s. The patient's condition stabilized, allowing for discharge on March 11, 2023, following evaluation by the attending physician. The patient returned for follow-up visits in the first and fourth months post-discharge, during which liver function, markers of myocardial damage, and platelet counts were found to have returned to normal levels. These findings suggest a favorable prognosis for the patient.

**Table 1:** Monitoring of organ functions.

Parameters	Day 1	Day 3	Day 7	Day 13
Echocardiogram				
LVEF (%)	20	38	42	43
LVID (cm)	6.2	6.0	4.3	3.7
Biochemical indicators				
hscTnI (ng/ml)	0.69	0.34	0.15	/
NT-proBNP (pg/ml)	5,682.0	814.8	292.5	29.4
ALT (IU/L)	5,358.2	4,240.3	961.4	189.5
AST (IU/L)	16,734.6	5,477.2	111.0	67.0
TBIL (µmol/L)	54.9	85.2	78.9	33.0

LVEF, left ventricular ejection fraction; LVID, left ventricularend-systolic dimension; hscTnI, high-sensitivity cardiac troponin I; NT-proBNP, N-terminal pro-B-type natriuretic peptide; ALT, alanine aminotransferase; AST, aspartate aminotransferase; TBIL, total bilirubin.

## Discussion

We report the case of a patient with rapidly progressive disease characterized by severe hemodynamic disturbances and multi-organ damage, specifically affecting the liver and kidneys. These manifestations, in conjunction with prodromal symptoms and evidence of myocardial injury, are consistent with a clinical diagnosis of FM. Upon admission, the patient underwent testing for antibodies to myocarditis-associated viruses. While positive viral serology does not confirm myocardial infection, it may indicate an interaction between the peripheral immune system and the source of the infection. Previous studies have suggested that viral serology has a limited role in diagnosing viral myocarditis due to the high prevalence of circulating IgG antibodies to cardiophilic viruses in the general population, even in the absence of viral heart disease (10). However, our patient tested positive for IgM antibodies to coxsackievirus B3 and echovirus, which may indicate a recent infection with these viruses, thereby supporting the diagnosis of viral myocarditis. The confirmation of viral myocarditis typically relies on endomyocardial biopsy (EMB) (4). However, in this case, EMB was not performed due to the patient's severe coagulopathy and the associated high risk of bleeding from invasive procedures. Furthermore, it is essential to rule out coronary artery disease and other cardiovascular conditions, such as hypertension and acute myocardial infarction, as well as extracardiac non-inflammatory disorders that could account for the clinical manifestations of FM (11). This is particularly important because these conditions may present with similar symptoms, including chest pain, and elevated levels of circulating biomarkers of myocardial injury, such as troponin and B-type natriuretic peptide (12).

In terms of aetiological treatment, there are currently no approved pathogen-directed or antiviral therapies for patients with viral myocarditis. Given the limited evidence supporting the efficacy of intravenous immunoglobulin therapy in this context, the International Society of Cardiology has refrained from making specific recommendations regarding its use. The pathogenesis of myocardial dysfunction in myocarditis is primarily attributed to a maladaptive hyperimmune response triggered by viral infection; thus, therapies aimed at modulating the immune response are considered potentially beneficial (13). In the case of our patient, and in the absence of a specific antiviral agent targeting coxsackievirus and echovirus, human immunoglobulin (PH4) was selected as an immunosupportive therapy. This choice is predicated on its content of neutralizing enterovirus antibodies, which may confer effectiveness against the implicated viral pathogens. Additionally, the patient received hormonal anti-inflammatory treatment to further support the management of the immune response. However, multicentre, placebo-controlled trials of intravenous immunoglobulin therapy in adult patients are necessary to establish its efficacy in individuals with biopsy-proven myocarditis.

Notably, the patient was admitted with cardiogenic shock and severe liver injury, which were believed to result from inadequate perfusion and venous congestion associated with the cardiogenic shock. This condition led to liver injury and failure, subsequently causing coagulopathy. The incidence of liver dysfunction in patients with acute heart failure has been reported to range from 20% to 30% (14). The coagulation indices indicated the presence of coagulation disorders, consistent with liver dysfunction, which included deficiencies in coagulation factors (such as fibrinogen and vitamin K-dependent factors), thrombocytopenia, and hyperfibrinolysis. The patient's coagulation parameters were characterized by decreased fibrinogen, decreased PLT, prolonged PT, and significantly elevated D-dimer levels, leading to a calculated score indicative of overt DIC. The question arises: is this patient's coagulation disorder due to liver failure or DIC? Notably, FVIII activity was measured at 176.5%, with an APTT of 35.6 s—neither of which supports a diagnosis of DIC. When liver function tests are markedly abnormal, it can be challenging to ascertain whether laboratory changes stem from liver disease, DIC, or a combination of both. The level of FVIII activity serves as a critical differentiating factor; FVIII is synthesized not only in the liver but also by extrahepatic endothelial cells. In patients with liver disease, increased levels of extrahepatic synthesis often result in normal or elevated FVIII activity. In contrast, patients with DIC typically exhibit significantly reduced levels of FVIII and other coagulation factors due to depletion resulting from coagulation overactivation (15, 16).

In patients with both abnormal liver function and coagulopathy, the question arises: do they require anticoagulation? Liver insufficiency is a recognized risk factor for venous thrombosis, and anticoagulation is indicated in patients with acute liver injury, even in the presence of prolonged PT and thrombocytopenia (17). Unfractionated heparin is often considered the optimal choice for anticoagulation due to its short half-life, reversibility, non-renal clearance, and ease of monitoring (18). Consequently, we administered a low dose of unfractionated heparin (300 U/h) for anticoagulant therapy and observed a sustained decrease in D-dimer levels. In patients with FM, managing coagulation disorders presents a significant challenge that necessitates careful consideration of treatment strategies. Anticoagulation is often emphasized in patients with liver dysfunction; however, those with acute liver injury face an increased risk of bleeding due to impaired synthesis of coagulation factors and platelet dysfunction. This risk can be exacerbated by anticoagulation therapy (19). To mitigate this, supplementation with FFP and specific clotting factors may help restore hemostatic balance in patients suffering from severe liver dysfunction secondary to FM. The use of FFP in patients with liver disease remains controversial due to its potential to exacerbate portal hypertension. Nevertheless, in non-cirrhotic patients, fibrinogen levels below 1 g/L are associated with an increased risk of bleeding, and maintaining levels above 1.2 g/L is recommended for those experiencing active bleeding (20). Our treatment protocol includes intermittent supplementation of FFP in small doses, alongside cryoprecipitate infusion, to maintain fibrinogen levels above 1.2 g/L and achieve a balance between anticoagulation and hemostasis. Clinically, we have observed that the combined use of FFP and cryoprecipitate is effective in addressing coagulopathy in patients with FM. This strategy not only reduces bleeding risks but also supports overall recovery by stabilizing hemostatic function. Additionally, attention must be given to platelet levels, which are crucial for hemostasis. The use of IABP support led to a mechanical destruction of platelets, resulting in a count of 18 × 10^9^ /L. Given that the IABP could not be temporarily withdrawn at that time, we evaluated the situation and decided to transfuse 1 unit of platelets. However, the platelet count subsequently decreased again, from 57 × 10^9^ /L to 19 × 10^9^ /L. After careful assessment, we opted to withdraw IABP support, and considering the low risk of bleeding, we did not proceed with additional platelet transfusions. As the patient's liver function gradually improved, we observed a corresponding rebound in platelet counts. Our treatment strategy, which involved simultaneous supplementation and anticoagulation, effectively corrected the patient's coagulation function, leading to a favorable prognosis.

## Conclusions

This case underscores the complexity of managing a patient with FM complicated by cardiogenic shock, severe liver injury, and coagulopathy. The therapeutic challenges in such critically ill patients necessitate an active search for the underlying etiology. It is essential to maintain a delicate balance within the coagulation system while implementing a combination of therapeutic regimens. This includes developing an optimal anticoagulation strategy and determining appropriate timing for interventions. Furthermore, alternative therapies must be considered, balancing the associated risks of bleeding and thrombosis. Such comprehensive management is crucial for improving patient outcomes in this intricate clinical scenario.

## Data Availability

The raw data supporting the conclusions of this article will be made available by the authors, without undue reservation.
